# Sex, Age, and Bodyweight as Determinants of Extracellular DNA in the Plasma of Mice: A Cross-Sectional Study

**DOI:** 10.3390/ijms20174163

**Published:** 2019-08-26

**Authors:** Ľubica Janovičová, Barbora Konečná, Lenka Vokálová, Lucia Lauková, Barbora Vlková, Peter Celec

**Affiliations:** 1 Institute of Molecular Biomedicine, Faculty of Medicine, Comenius University, Sasinkova 4, 811 08 Bratislava, Slovakia; 2 Institute of Physiology, Faculty of Medicine, Comenius University, Sasinkova 2, 813 72 Bratislava, Slovakia; 3 Department of Biomedicine, University of Basel, Hebelstrasse 20, 4031 Basel, Switzerland; 4 Center for Biomedical Technology, Department for Health Sciences and Biomedicine, Danube University, 3500 Krems, Austria; 5 Institute of Pathophysiology, Faculty of Medicine, Comenius University, Sasinkova 4, 81108 Bratislava, Slovakia; 6 Department of Molecular Biology, Faculty of Natural Sciences, Comenius University, Mlynská dolina, Ilkovičova 6, 842 15 Bratislava, Slovakia

**Keywords:** cell-free DNA, nuclease activity, aging, obesity, gender differences

## Abstract

Extracellular DNA (ecDNA) is studied as a possible biomarker, but also as a trigger of the immune responses important for the pathogenesis of several diseases. Extracellular deoxyribonuclease (DNase) activity cleaves ecDNA. The aim of our study was to describe the interindividual variability of ecDNA and DNase activity in the plasma of healthy mice, and to analyze the potential determinants of the variability, including sex, age, and bodyweight. In this experiment, 58 adult CD1 mice (41 females and 31 males) of a variable age (3 to 16 months old) and bodyweight (females 25.7 to 52.1 g, males 24.6 to 49.6 g) were used. The plasma ecDNA was measured using a fluorometric method. The nuclear ecDNA and mitochondrial ecDNA were quantified using real-time PCR. The deoxyribonuclease activity was assessed using the single radial enzyme diffusion method. The coefficient of variance for plasma ecDNA was 139%, and for DNase 48%. Sex differences were not found in the plasma ecDNA (52.7 ± 73.0 ηg/mL), but in the DNase activity (74.5 ± 33.5 K.u./mL for males, and 47.0 ± 15.4 K.u./mL for females). There were no associations between plasma ecDNA and bodyweight or the age of mice. Our study shows that the variability of plasma ecDNA and DNase in adult healthy mice is very high. Sex, age, and bodyweight seem not to be major determinants of ecDNA variability in healthy mice. As ecDNA gains importance in the research of several diseases, it is of importance to understand its production and cleavage. Further studies should, thus, test other potential determinants, taking into account cleavage mechanisms other than DNase.

## 1. Introduction

Extracellular DNA (ecDNA) is DNA outside of cells. The presence of ecDNA in blood was discovered by Mandel and Metais in 1948 [1]. The ecDNA is proinflammatory [2] and procoagulatory [3]. Apoptosis and necrosis are the major sources of ecDNA [4]. EcDNA is used as a biomarker in non-invasive prenatal diagnostics [5] and oncology [6]. In addition, donor DNA can be detected in the blood of the transplant recipient, suggesting that ecDNA could be a biomarker of transplant rejection [7,8]. EcDNA is also a therapeutic target. In several diseases, it has been shown that ecDNA leads to inflammation and a release of more ecDNA—this vicious circle can be stopped by the removal of ecDNA, as shown for sepsis [9], hepatorenal injury [10], and metabolic syndrome [11,12].

The knowledge about the normal ecDNA concentration values and the variability of ecDNA is limited. Currently, there are no standard physiological values for ecDNA in plasma. A recent study evaluating human plasma ecDNA showed that both nuclear and mitochondrial DNA vary within a range of over three orders of magnitude [13]. Regarding the technical variability, the amount of ecDNA found in body fluids depends on the processing of samples, and on the technique used for DNA quantification. It has been shown that the extraction efficiency from plasma has a coefficient of variance of approximately 29% [14]. The discordant methods of sample processing and quantification make a comparison of ecDNA concentrations between studies difficult [15]. Apart from technical variability, there is also a high biological variability of plasma ecDNA. This is best described for fetal DNA circulating in maternal blood [16]. Deoxyribonuclease-I (DNase I) does not affect the fragmentation of ecDNA in plasma [17]. The pattern of fragmentation of ecDNA was described in mice deficient in DNase I and DNase 1L3. Mice without DNase 1l3 have more longer fragments of ecDNA [18]. However, if mice are deficient for both DNase I together with DNase 1L3, and their neutrophils are stimulated, they die because of the inability to cleave ecDNA in blood [19].

Interindividual differences in ecDNA can be caused by differences in the release of ecDNA, its cleavage, or protection against the cleavage. Deoxyribonucleases (DNases) are able to cleave plasma ecDNA [20]. Obesity inducing the release of ecDNA from adipocytes makes body weight one of the candidate determinants of ecDNA [11]. Aging is associated with an increased production of neutrophil extracellular traps—a major source of ecDNA [21]. Women are more prone to autoimmune diseases associated with anti-DNA antibodies [22]. This suggests that the concentration of ecDNA in females could be higher, although the mechanisms are unclear. Understanding the variability of ecDNA could be very helpful to overcome the diagnostic limitations of ecDNA, but it could also shed light on the role of ecDNA in the pathogenesis of inflammatory diseases. The aim of this study is to describe the variability of ecDNA and DNase, and to analyze its potential determinants. Our hypothesis was that older females with a higher bodyweight have higher ecDNA and lower DNase in their plasma.

## 2. Results

An analysis of variability revealed high coefficients of interindividual variations for total ecDNA (Coefficient of Variance (CV) = 139%), nuclear DNA (ncDNA; CV = 50%), and mitochondrial (mtDNA; CV = 149%). The total ecDNA minimum is 100-fold lower than the maximum concentration. The maximum ncDNA and mtDNA concentrations were 10-fold and 1000-fold higher than the minimum concentrations, respectively. For the DNase activity, the maximum value was 7.5-fold higher than the minimum. Based on the 5% and 95% percentile, the normal values of the total ecDNA, ncDNA, mtDNA, and DNase activity were determined. These are solely specific for the type of sample processing described here. The normal values for the total ecDNA in the plasma were 5 to 266 ng/mL. For the ecDNA fractions, normal values were also determined. The normal values for plasma mtDNA were 6.5 × 10^3^ to 6.4 × 10^5^ GE/mL, and for the plasma ncDNA they were from 1.6 × 10^3^ to 7.5 × 10^3^ GE/mL of plasma. The mtDNA copy number is, thus, approximately 4 to 85 per cell. The normal values of DNase activity in the plasma were from 32 to 125 K.u./mL (Table 1).

**Figure 1:**
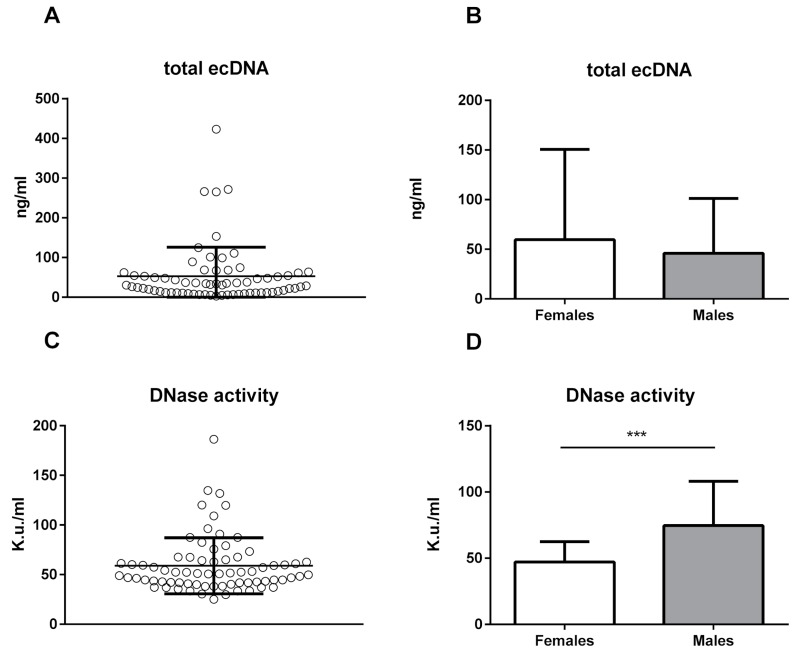
(**a**) Total extracellular DNA (ecDNA; *n* = 57) and (**b**) total ecDNA divided by sex (females *n* = 34, males *n* = 23) (*t*-test; *p* = 0.52, *t* = 0.64) and (**c**) deoxyribonuclease (DNase) activity (*n* = 72) and (**d**) DNase activity divided by sex (females *n* = 41, males *n* = 31) (*t*-test; *p* < 0.001, *t* = 4.65). DNase activity is expressed in Kunitz units (K.u.) per ml of plasma. The results are presented as mean + standard deviation. *p*-value is indicated as *** for *p* ˂ 0.001.

**Figure 2:**
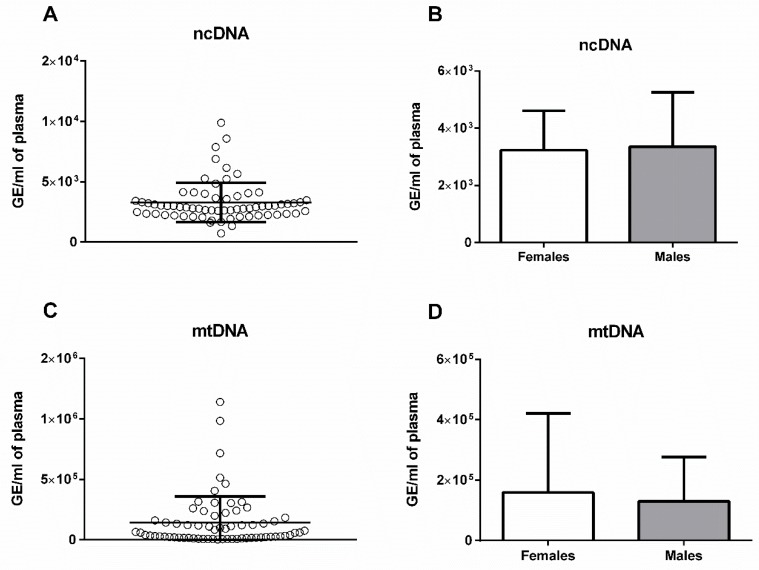
(**a**) Nuclear extracellular DNA (ncDNA; *n* = 57) and (**b**) nuclear extracellular DNA divided by sex (females *n* = 34, males *n* = 23; *t*-test; *p* = 0.26, *t* = 1.14) and (**c**) mitochondrial extracellular DNA (mtDNA; *n* = 57), and (**d**) mitochondrial extracellular DNA divided by sex (females *n* = 34, males *n* = 23; *t*-test; *p* = 0.41, *t* = 0.83). The extracellular DNA of both a nuclear and mitochondrial origin is expressed in genome equivalents (GE) per ml of plasma. The results are presented as mean + standard deviation.

Sex seems not to be a major determinant of plasma ecDNA variability in mice. There were no sex differences in the ecDNA concentrations in the total ecDNA (*t*-test; *p* = 0.52, *t* = 0.64; Figure 1A,B), ncDNA (*t*-test; *p* = 0.26, *t* = 1.14), or mtDNA (*t*-test; *p* = 0.41, *t* = 0.83; Figure 2). A statistically significant sex difference was found in the DNase activity—the male mice had a higher DNase activity by 59% (Figure 1D; *p* < 0.001, *t* = 4.65).

The correlation analysis revealed that the total ecDNA was positively correlated with ncDNA (*p* ˂ 0.001, Pearson’s *r* = 0.66) and strongly with mtDNA (*p* ˂ 0.001; Pearson’s *r* = 0.90). NcDNA was also positively correlated with mtDNA (*p* ˂ 0.001; Pearson’s *r* = 0.63). The DNase activity did not correlate with the total ecDNA (n.s.; Pearson’s *r* = −0.11). No association was found between the DNase activity and ncDNA (n.s.; Pearson’s *r* = −0.02) or mtDNA (n.s.; Pearson’s *r* = −0.02). The bodyweight of the mice did not correlate with the total ecDNA (n.s.; Pearson’s *r* = 0.01), ncDNA (n.s.; Pearson’s *r* = 0.07), or mtDNA (n.s.; Pearson’s *r* = −0.01). Similarly, age did not correlate with the total ecDNA (n.s.; Pearson’s *r* = 0.03), ncDNA (n.s.; Pearson’s *r* = 0.04), and mtDNA (n.s.; Pearson’s *r* = 0.04; Table 2).

## 3. Discussion

In this cross-sectional study on healthy mice, we confirmed a very high variability in ecDNA and DNase activity. As ecDNA and its cleavage are likely involved in the pathogenesis of several diseases, it is of importance to analyze the determinants of this variability. However, using correlation analysis, in our study, we found no association of either ecDNA or DNase with the age or bodyweight of the mice. Similarly, no sex differences were found for ecDNA or its fractions—ncDNA and mtDNA. The only sex difference was found in the DNase activity, which was higher in male mice, confirming previous reports [23]. Thus, to explain the variability of ecDNA, cleavage mechanisms other than the action of DNases should be taken into account. These include the phagocytic activity of monocytes, cleavage by the liver, or excretion via kidney [24,25,26].

There are many age-related diseases that are associated with high concentrations of ecDNA, such as autoimmune diseases [27], myocardial infarction [28], obesity [11,29], and others. It has already been published that aging is associated with increased levels of ecDNA in humans [30]. Dying cells release ecDNA to the extracellular space, and this ecDNA can activate toll-like receptors and induce an immune system response [31]. Neutrophils can die while releasing their DNA. Neutrophil extracellular trap production is increased in aged mice [32]. Nevertheless, in our study in healthy mice, age did not affect ecDNA or DNase in the plasma.

Regarding ecDNA, there is one published study investigating sex differences in ecDNA concentration. Men were shown to have a higher nuclear ecDNA concentration in comparison to women, but there were no differences in ecDNA originating from the mitochondria [13]. In our study, no such sex differences in ecDNA were found. In comparison to humans, mice have a much higher DNase activity in the plasma. Whether this affects the interpretability of the results of the experiments on animal models of diseases related to ecDNA is unknown. From the available literature, the variability in ecDNA and DNase activity is high [23]. This was confirmed by our results in mice. However, except for the sex difference in the DNase, the determinants of this variability could not be identified. Other factors should be studied in the future. Magnesium and calcium ions, for example, are needed for DNases to be active, and can be one of the determinants of DNase activity that contribute to variability [33]. 

One of the proposed determinants of ecDNA is physical activity. DNase activity and ecDNA were shown to increase in response to high-intensity exercise [34]. Circadian biorhythms affect DNA damage and apoptosis [35]. Similarly, in females, the variability of ecDNA or DNase activity could be influenced by the estrous cycle. The hormonal changes might be relevant in autoimmune diseases related to ecDNA and their animal models [36]. Revealing the determinants of ecDNA variability both in humans and in mice, might help to understand the physiology and the pathophysiology related to ecDNA.

One important aspect not included in our study is technical variability. It has been shown that even the isolation of ecDNA contributes considerably to the overall variability in the outcome [14]. The optimization of protocols is crucial in achieving accurate and reproducible results. The centrifugation speed and time differ between studies, which makes it difficult to compare the results. The most commonly used protocol is two-step centrifugation, which removes apoptotic bodies and some microvesicles [37]. The choice of blood collection method is of importance. Serum is not suitable for ecDNA quantification, because of the release of genomic DNA from cells during coagulation [38]. EDTA plasma, on the other hand, cannot be used for the measurement of DNase activity, as it inhibits DNase I [39]. So, in our study, we have collected blood into EDTA and heparin to use plasma for DNA and DNase measurements, respectively. However, a further reduction of the technical variability, especially in the quantification of nuclear and mitochondrial DNA, is surely needed. 

In conclusion, for future experiments, it is important to consider the high interindividual variability of ecDNA in healthy animals, which is independent of body weight, age, or sex. The observed sex difference in the DNase activity needs to be taken into consideration, especially if confirmed in other strains and species. In addition, the underlying endocrine or genetic mechanisms should be clarified. The major contributors to the biological variability of ecDNA are, however, yet to be identified. 

## 4. Materials and Methods 

### 4.1. Animal Procedures

The CD-1 mice were purchased from Anlab (Prague, Czech Republic). Both female (*n* = 35) and male (*n* = 23) healthy mice of varying age (7.5 ± 4 months) and of varying weights (females 34.1 ± 7.4 g and males 40.7 ± 6.2 g) had ad-libitum access to food and water. The animals were maintained in a temperature-controlled and light-controlled room with a 12-h light/dark cycle. Blood was collected from the retro-orbital plexus from mice in isoflurane anesthesia (AbbVie, Bratislava, Slovakia) into heparin and EDTA microvettes (Sarstedt, Nümbrecht, Germany), and centrifuged at 2000× *g* for 10 min at 4 °C. The mice were sacrificed by cervical dislocation. The plasma was stored at −20 °C until further analyses. All of the methods and procedures were conducted in accordance with Slovak legislation. The animals were housed and procedures were conducted in compliance with the Ethics committee of Institute of Molecular Biomedicine, Comenius University in Bratislava (Ro-536/18-221/3; date: 26 March 2018). 

### 4.2. DNA Isolation and Quantification

DNA was isolated from the EDTA plasma using the QIAamp DNA Blood Mini kit (Qiagen, Hilden, Germany). The isolated DNA was quantified using a Qubit 3.0 fluorometer and Qubit dsDNA high sensitivity assay (Thermo Fisher Scientific, Waltham, MA, USA). The fractions of nuclear and mitochondrial DNA were estimated using real-time PCR on the Mastercycler realplex 4 (Eppendorf, Hamburg, Germany) with Sso Advanced Universal SYBR Green Supermix (Bio Rad Laboratories, Hercules, CA, USA). The following PCR program was used: one cycle of 98 °C for 3 min, 40 cycles of 98 °C for 15 s for denaturation, 60 °C for 30 s for annealing, and 72 °C for 30 s for extension. Primers were designed for the amplification of mouse mtDNA (F: 5′-CCCAGCTACTACCATCATTCAAGT-′, R: 5′-GATGGTTTGGGAGATTGGTTGATGT-3′) [40] and ncDNA (F: 5′-TGTCAGATATGTCCTTCAGCAAGG-3′, R: 5′-TGCTTAACTCTGCAGGCGTATG-3′) [41]. The quantified ecDNA is expressed in genome equivalents (GE).

### 4.3. DNase Activity

Plasma anticoagulated with heparin was used for the determination of DNase activity. The DNase activity was measured using the modified single radial enzyme-diffusion assay with the GoodView fluorescent dye [42]. Agarose gels (1%, 20 mM Tris-HCl, ph 7.5, 2 mM MgCl_2_, 2 mM CaCl_2_) containing DNA isolated from chicken livers (0.5 mg/mL of DNA) were visualized using iBOX (Vision works LP Analysis Software, UVP, Upland, CA, USA), and the radius of the circles were measured and compared to the calibration curve from the DNase standards. The DNase activity was recalculated and expressed in Kunitz units (K.u.).

### 4.4. Statistical Analysis

The results were analyzed using GraphPad Prism 6 (La Jolla, CA, USA). For the evaluation of the sex differences, the student *t*-test was used. Correlation analyses were conducted with the Pearson correlation test. The test results of *p* ˂ 0.05 were considered statistically significant. 

## Figures and Tables

**Table 1:** Descriptive statistics of the measured variables, namely: bodyweight, age, plasma extracellular DNA (ecDNA), and deoxyribonuclease (DNase) activity in all of the mice combined.

	Bodyweight (g)	Age (Days)	Total ecDNA (ng/mL)	ncDNA (GE/mL)	mtDNA (GE/mL)	DNase Activity (K.u./mL)
Mean	38.06	226	52.7	3.3 × 10^3^	1.4 × 10^5^	59.0
Standard Error	0.72	14	8.8	2.0 × 10^2^	2.6 × 10^4^	3.4
Median	38.53	195	31.1	2.9 × 10^3^	6.0 ×10^4^	51.0
Standard Deviation	6.16	122	73.0	1.6 × 10^3^	2.1 × 10^5^	28.4
Minimum	24.62	99	2.9	7.2 × 10^2^	3.1 × 10^3^	25.1
Maximum	52.11	468	423.2	9.9 × 10^3^	1.1 × 10^6^	186.4
Coefficient of Variance	16%	54%	139%	50%	149%	48%

**Table 2:** Correlation matrix describing the relationships between the analyzed variables, namely: bodyweight, age, total extracellular DNA (ecDNA), nuclear ecDNA (ncDNA), mitochondrial ecDNA (mtDNA), and deoxyribonuclease (DNase) activity. The data in the table are presented as Pearson’s correlation coefficients with *p*-values indicated as *p* ˂ 0.001 for ***.

	Bodyweight	Age	Total ecDNA	ncDNA	mtDNA	DNase Activity
Bodyweight		0.60 ***	0.01	0.07	−0.01	0.19
Age	0.60 ***		0.03	0.04	0.03	−0.11
Total ecDNA	0.01	0.03		0.66 ***	0.90 ***	−0.11
ncDNA	0.07	0.04	0.66 ***		0.63 ***	−0.02
mtDNA	−0.01	0.03	0.90 ***	0.63 ***		−0.02
DNase activity	0.19	−0.11	−0.11	−0.02	−0.02	

## Abbreviations

DNase	Deoxyribonuclease
mtDNA	Mitochondrial DNA
ncDNA	Nuclear DNA
ecDNA	Extracellular DNA
